# Prognostic Value of Plaque Burden Beyond Stenosis Severity in Patients with Stable Chest Pain Undergoing Coronary CT Angiography

**DOI:** 10.3390/medsci14060648

**Published:** 2026-10-08

**Authors:** Ana Ezponda, José Marlon Rodríguez, Miguel Sogbe, Patricia Boldó, Ariana Álvarez, Juan P. de-Torres, Nahikari Salteráin, Juan José Gavira, Gorka Bastarrika

**Affiliations:** 1 Radiology Department, Clínica Universidad de Navarra, 31008 Pamplona, Spain; aezponda@unav.es (A.E.); jrodriguezortega@unav.es (J.M.R.); 2 Liver Unit, Clínica Universidad de Navarra, 31008 Pamplona, Spain; msogbe@unav.es; 3 Faculty of Medicine, University of Navarra, 31009 Pamplona, Spain; pboldosorri@alumni.unav.es; 4 Cardiology Department, Clínica Universidad de Navarra, 31008 Pamplona, Spain; aalvareza@unav.es (A.Á.); nsalterain@unav.es (N.S.); jjgavira@unav.es (J.J.G.); 5 Pneumology Department, Clínica Universidad de Navarra, 31008 Pamplona, Spain; jpdetorres@unav.es

**Keywords:** coronary artery disease, CAD-RADS, plaque burden, major adverse cardiovascular events, coronary CT angiography

## Abstract

**Introduction:** Coronary computed tomography angiography (CCTA) is a noninvasive test for suspected coronary artery disease (CAD), but the prognostic value of integrating plaque burden into CAD-RADS 2.0 has not been fully evaluated. We aimed to assess whether the high-risk CAD-RADS 2.0 category, which combines stenosis severity and segment involvement score (SIS), provides prognostic information beyond stenosis severity in patients with stable chest pain. **Methods:** We retrospectively included 563 consecutive patients with stable chest pain who underwent CCTA between 2017 and 2022. CAD-RADS 1.0 categories and CAD-RADS 2.0 plaque burden grades were assigned by experienced radiologists. High-risk CAD-RADS 2.0 was defined as CAD-RADS ≥ 3 or any CAD-RADS with P-SIS ≥ 3. The primary endpoint was major adverse cardiovascular events (MACEs). Given the limited number of events, multivariable analysis was restricted to clinically justified parsimonious Cox proportional hazards models. A model including age, sex, and significant stenosis was compared with a model including age, sex, and the high-risk CAD-RADS 2.0 category. A formally nested extended model additionally included severe-to-extensive plaque burden. **Results:** Over a median follow-up of 51.2 months, 41 patients (7.3%) experienced MACEs. Among patients with non-obstructive CAD (CAD-RADS 1–2), 29 of 477 (6.1%) experienced MACEs, and 85.4% of these events occurred in patients with severe or extensive plaque burden (P3–P4). After adjustment for age and sex, significant stenosis was not associated with MACEs, whereas the high-risk CAD-RADS 2.0 category was independently associated with MACEs (HR 2.88, 95% CI 1.07–7.75; *p* = 0.036). Addition of plaque burden to a model including age, sex, and significant stenosis increased the Harrell C-index from 0.760 to 0.772 and significantly improved model fit (likelihood-ratio χ^2^ = 5.41; *p* = 0.020). In the extended model, plaque burden remained independently associated with MACEs (HR 2.81, 95% CI 1.10–7.19; *p* = 0.031). **Conclusions:** In patients with stable chest pain undergoing CCTA, the high-risk CAD-RADS 2.0 category was independently associated with MACEs. Formal incremental analyses demonstrated that plaque burden provided prognostic information beyond age, sex, and significant stenosis. These findings support the prognostic relevance of integrating plaque burden into CCTA-based risk classification but require confirmation in larger prospective and externally validated cohorts.

## 1. Introduction

Coronary artery disease (CAD) remains a leading cause of morbidity and mortality worldwide, highlighting the need for accurate risk stratification to optimize patient outcomes [1]. Coronary Computed Tomography Angiography (CCTA) has become the first-line noninvasive imaging modality for the assessment of coronary arteries in patients with chest pain and suspected CAD [2,3]. Owing to its high sensitivity, CCTA is particularly well suited to reliably rule out obstructive coronary artery stenosis, and multiple studies, including randomized clinical trials, have demonstrated its prognostic value for predicting major adverse cardiovascular events (MACEs) [4].

The Coronary Artery Disease–Reporting and Data System (CAD-RADS 1.0), introduced in 2016, was designed to standardize CCTA reporting and quickly gained widespread adoption after its prognostic relevance was demonstrated [5]. This classification focuses primarily on the degree of luminal stenosis in the epicardial coronary arteries. Beyond identifying obstructive disease, CCTA also provides comprehensive information on nonobstructive CAD [6]. Large cohort studies, such as CONFIRM and SCOT-HEART, have shown that extensive nonobstructive plaque burden significantly increases the risk of myocardial infarction and death. Data suggest that the extension of nonobstructive plaque plays an important role in the development of acute cardiovascular events and is a predictor of all-cause mortality [7,8].

In 2022, the updated CAD-RADS 2.0 reporting system was developed through a consensus of experts from various European and American societies of radiology and cardiology [9]. This updated system expands on the original framework by incorporating additional parameters, most notably the overall burden of coronary atherosclerosis. This is represented by the “P” modifier, which can be quantified by the number of segments involved with plaque, referred to as the segment involvement score (SIS) [10].

CAD-RADS 2.0 considers patients with moderate-to-severe stenosis (CAD-RADS 3–5) and/or high plaque burden (P3–P4) to be at increased risk of cardiovascular events. However, the incremental value of adding plaque burden to stenosis severity has not been formally assessed in patients with chronic chest pain and no prior CAD.

Therefore, the aim of this study was to evaluate whether the high-risk CAD-RADS 2.0 classification, which integrates coronary plaque burden measured by SIS with stenosis severity, provides prognostic information beyond stenosis severity in patients with stable chest pain and suspected CAD undergoing CCTA.

## 2. Materials and Methods

### 2.1. Population

The study included 1139 consecutive adults (≥18 years) with stable chest pain who were referred to the Cardiology Department of our hospital and underwent CCTA between September 2017 and April 2022. CCTAs were ordered to rule out CAD.

**Figure 1:**
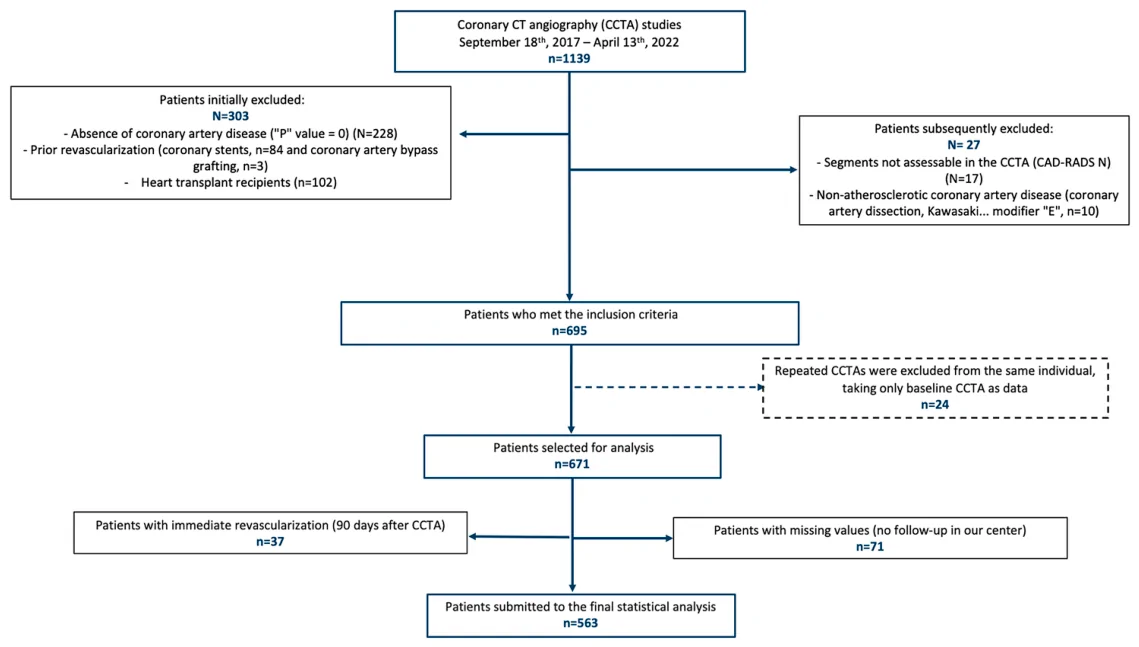
Patient flowchart. Abbreviations: CAD-RADS, Coronary Artery Disease–Reporting and Data System; CCTA, coronary computed tomography angiography.

Patients without CAD (CAD-RADS = 0) and thus without a “P” value, patients with a history of coronary revascularization (percutaneous or surgical), and heart transplant recipients were initially excluded. In addition, CCTAs with non-assessable segments (CAD-RADS N) and patients with non-atherosclerotic coronary disease (CAD-RADS 2.0 modifier “E”) were excluded (Figure 1). After applying these criteria, 671 consecutive outpatients met the inclusion criteria. For the main statistical analyses, we further excluded individuals who underwent early revascularization, defined as percutaneous or surgical revascularization within 90 days after CCTA, to allow a clearer assessment of the prognostic impact of CCTA findings on MACEs without the confounding effect of early therapeutic interventions. To assess the potential impact of this exclusion, a prespecified sensitivity analysis reincorporated these patients and repeated the time-to-event models for subsequent clinical outcomes. After applying the primary criteria, the final study cohort consisted of 563 patients.

The study was conducted in accordance with the Declaration of Helsinki, and the institutional ethics committee waived the requirement for specific individual informed consent owing to the retrospective observational design based on routinely collected data.

### 2.2. CCTA Acquisition

All examinations were performed with a third-generation dual-source CT scanner (SOMATOM Force, Siemens Healthineers, Forchheim, Alemania) following a standardized protocol. Before the angiographic acquisition, a non-contrast, ECG-synchronized scan was obtained in all patients for coronary calcium quantification, using prospective gating or single-beat acquisition. The study range extended from the tracheal carina to the diaphragm dome and was reconstructed with specific quantification filters (Qr36). Prospective or retrospective acquisition was chosen based on cardiac rhythm variability. A triphasic injection protocol was used for all studies [11].

For each patient, optimal diastole and/or systole phases were automatically reconstructed, with additional reconstructions generated in 5% increments within the maximum radiation dose window. Images were reconstructed with a slice thickness of 0.6 mm and an increment of 0.4 mm, using iterative reconstruction algorithms (advanced modeled iterative reconstruction, ADMIRE, level 3), a specific reconstruction filter (Bv40), and a field of view adjusted to the heart. Both coronary calcium and angiographic datasets were transferred to a dedicated cardiac post-processing workstation (Syngo.via, Siemens Healthineers).

### 2.3. CCTA Interpretation

All CCTAs were evaluated by two cardiothoracic radiologists, with 22 and 5 years of experience (GB, AE), for CAD-RADS 1.0 and CAD-RADS 2.0 classifications. Segmental plaque analysis of all segments ≥ 2 mm in diameter was performed visually according to the American Heart Association (AHA) 16-segment model [12]. Readers were blinded to subsequent clinical outcomes. Discrepancies in CAD-RADS category or plaque burden assessment were resolved by consensus. Interobserver and intraobserver reproducibility of CAD-RADS classification and SIS was not formally assessed in this study. Although both measures have demonstrated acceptable reproducibility in previous investigations, we cannot exclude some degree of measurement variability.

The degree of coronary stenosis was categorized as 0–24% (normal/minimal), 25–49% (mild), 50–69% (moderate), and ≥70% (severe). Plaque burden was assessed using the segment involvement score (SIS) [9]. SIS was defined as the total number of coronary segments with detectable plaque, either calcified or non-calcified. For each of the 16 segments, a score of 1 was assigned if any plaque was present and 0 if absent, and the SIS for each patient was obtained by summing individual segment scores. Based on the CAD-RADS 2.0 grading system, patients were assigned to one of four plaque burden categories (P1–P4): P1 (mild, SIS ≤ 2), P2 (moderate, SIS 3–4), P3 (severe, SIS 5–7), and P4 (extensive, SIS ≥ 8) [9].

For subsequent analyses, coronary stenosis according to CAD-RADS was dichotomized as non-obstructive (CAD-RADS 1–2) or obstructive (CAD-RADS 3–5). Plaque burden based on P-SIS was similarly grouped as mild-to-moderate (P1–P2) or severe-to-extensive (P3–P4). In line with CAD-RADS 2.0 recommendations, patients were then reclassified into two risk categories according to the extent of CAD defined by P-SIS. Individuals with CAD-RADS ≥ 3 or any CAD-RADS category with P ≥ 3 were classified as high-risk [9].

### 2.4. Study Variables and Outcomes

Demographic characteristics, cardiovascular risk factors, cardiovascular history, relevant comorbidities, and usual medication were collected at time of the CCTA exam. Clinical data were documented based on patient-reported history.

The primary outcome was the occurrence of major adverse cardiovascular events (MACEs), defined as hospitalization for heart failure, acute myocardial infarction, unstable angina, stroke, or cardiovascular death. Follow-up data were obtained through systematic review of electronic medical records. All clinical events included in the study endpoints were identified and adjudicated by an experienced clinician who was blinded to the baseline CCTA findings and CAD-RADS classification. For each patient, time-to-event was calculated from the date of CCTA to the first MACE, death, or the date of the last available clinical report, whichever occurred first.

### 2.5. Statistical Analysis

#### 2.5.1. Descriptive Analysis

Descriptive analysis of the sample (baseline characteristics) was performed. Categorical variables are expressed as numbers and relative frequencies as percentages. Clinical and pharmacological variables were categorized as present (=1) or absent (=0). Smoking was categorized as never smokers (=0) and current or former smokers (=1). The distribution of quantitative variables was assessed using the Kolmogorov–Smirnov test. Normally distributed variables are reported as mean and standard deviation, whereas non-normally distributed variables are expressed as median and interquartile range (IQR). Between-group differences (patients with vs. without MACEs) were evaluated using the chi-square test for categorical variables and the Student t test or the Mann–Whitney U test for continuous variables, as appropriate.

#### 2.5.2. Associations of the Different Variables with MACE

Univariable Cox proportional hazards regression models were first used to assess the association between clinical, pharmacological, and CCTA variables and MACEs. Given the limited number of outcome events, multivariable analyses were restricted to parsimonious sets of clinically justified covariates. Covariate selection was based on clinical and prognostic relevance and was independent of statistical significance in the univariable analyses. No automated or stepwise variable-selection procedure was used.

Age and sex were included as core demographic prognostic factors. Significant stenosis was included to represent the conventional stenosis-based CCTA assessment, whereas severe-to-extensive plaque burden and the high-risk CAD-RADS 2.0 category were included to assess the prognostic contribution of plaque burden and of the integrated classification combining stenosis severity and plaque burden. Covariate selection was based on clinical and prognostic relevance and was independent of statistical significance in the univariable analyses. No automated or stepwise variable-selection procedure was used. Three primary Cox proportional hazards models were fitted. Model 1 included age, sex, and significant stenosis. Model 2 included age, sex, and severe-to-extensive plaque burden. Model 3 included age, sex, and the high-risk CAD-RADS 2.0 category.

#### 2.5.3. Assessment of Incremental Prognostic Value

Because the three primary parsimonious models included alternative CCTA predictors and were not nested, their discrimination was compared descriptively using Harrell’s C-index and the Akaike information criterion. To formally assess the incremental prognostic value of plaque burden, a reference Cox model including age, sex, and significant stenosis was compared with a nested extended model additionally including severe-to-extensive plaque burden. The nested models were compared using the likelihood-ratio test.

#### 2.5.4. Sensitivity Analysis

Three prespecified sensitivity analyses were additionally performed to assess the robustness of the primary findings. The first analysis considered only coronary ischemic events, defined as unstable angina and myocardial infarction. The second sensitivity analysis evaluated MACEs, defined as the composite of cardiovascular death, myocardial infarction, and unstable angina. The third analysis evaluated a broader atherosclerotic cardiovascular endpoint including coronary ischemic events (unstable angina or myocardial infarction), ischemic stroke, and cardiovascular death. Because of the relatively low number of events, additional sensitivity analyses were adjusted only for age and sex. Cox proportional hazards models were constructed using the same analytical approach as the primary analysis.

To evaluate the potential selection bias introduced by excluding patients undergoing coronary revascularization within 90 days after CCTA, another sensitivity analysis reincorporating these patients into the study cohort was performed. Patients were therefore followed for subsequent clinical events using the original MACE definition and time-to-event approach. Cox proportional hazards models were repeated before and after adjustment for age and sex.

Hazard ratios (HRs) and 95% confidence intervals (CIs) were reported for all models. Kaplan–Meier curves for the occurrence of MACEs were generated. All statistical analyses were performed using R version 4.4.0 and SPSS version 25.0 (IBM Corp., Armonk, NY, USA). A two-sided *p* value < 0.05 was considered statistically significant.

## 3. Results

### 3.1. Baseline Characteristics of the Sample

The final cohort included 563 patients (64.7% men) with suspected stable CAD. During a median follow-up of 51.2 months (IQR, 43.4–68.3), 41 patients (7.3%) experienced at least one component of the composite endpoint. The individual components were heart-failure hospitalization in 20 patients, unstable angina in 11, myocardial infarction in 6, ischemic stroke in 10 and cardiovascular death in 1. Because some patients experienced more than one event, component counts are not mutually exclusive.

Patients who developed MACEs were older than those without events (median age, 74 [IQR 68.5–81.5] vs. 62 [IQR 53–69] years; *p* < 0.001) and were more often women (58.5% vs. 33.5%; *p* < 0.001). MACEs occurred more frequently in patients with chronic obstructive pulmonary disease (COPD), hypertension, diabetes mellitus, chronic kidney disease, and a history of arrhythmias, whereas no significant differences were observed for smoking status, obesity, dyslipidemia, obstructive sleep apnea, or hyperuricemia (Table 1). Patients who developed MACEs were also more likely to receive antihypertensive drugs, beta-blockers, diuretics, antiplatelet agents, anticoagulants, antiarrhythmics, and nitrates (Table 2).

### 3.2. CCTA Findings

In the final sample, CAD-RADS categories were distributed as follows: CAD-RADS 1 in 351 patients (62.3%), CAD-RADS 2 in 126 (22.4%), CAD-RADS 3 in 44 (7.8%), CAD-RADS 4 in 32 (5.7%), and CAD-RADS 5 in 10 (1.8%). Plaque burden according to SIS was graded as P1 in 135 individuals (24%), P2 in 105 (18.7%), P3 in 84 (14.9%), and P4 in 239 (42.5%). Overall, 86 patients (15.3%) had obstructive CAD (CAD-RADS 3–5), while 325 (57.7%) were classified as a high-risk according to CAD-RADS 2.0 (Table 3).

**Figure 2:**
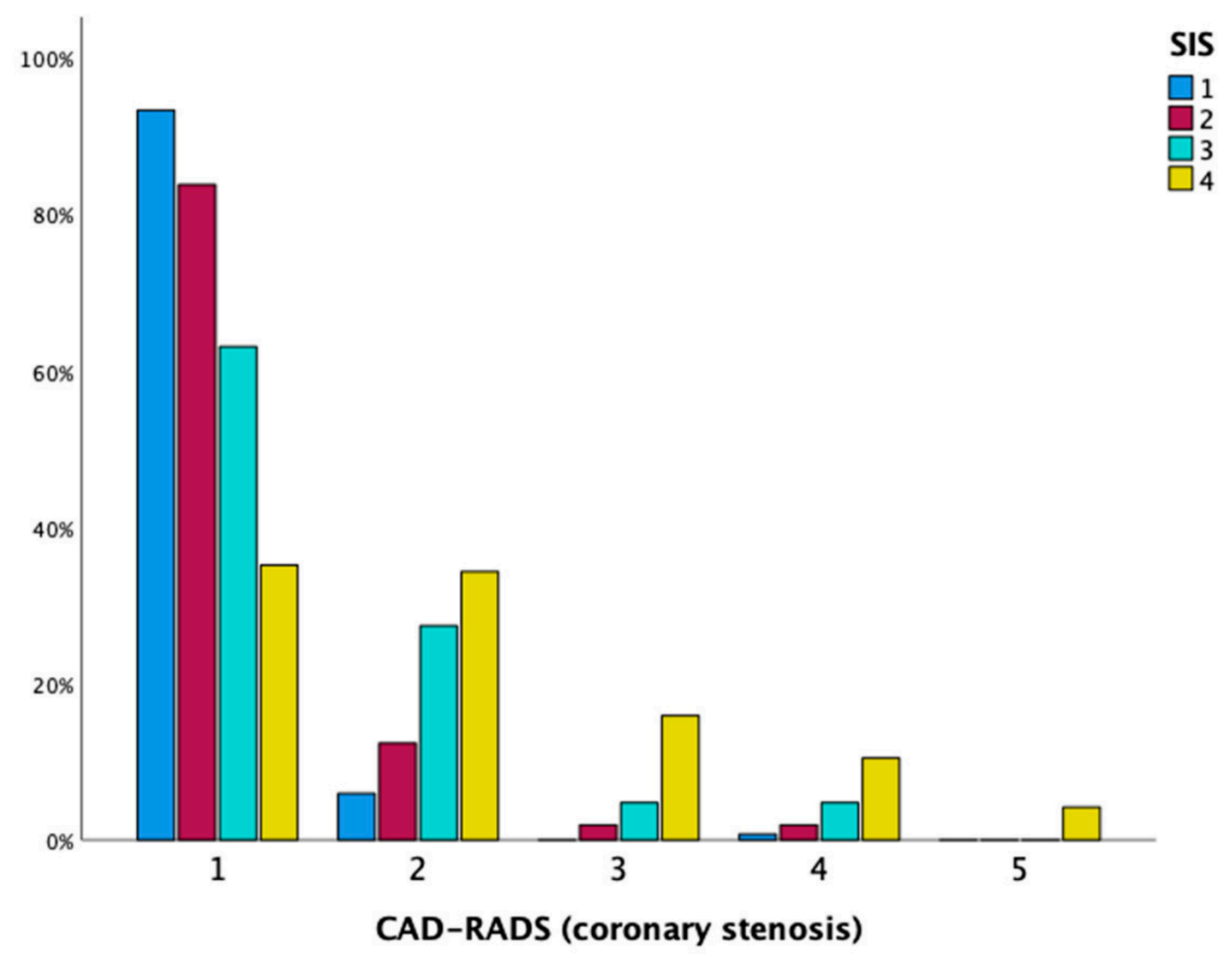
Association of coronary artery stenosis with atherosclerotic plaque burden. The ordinate axis is labeled as “Percentage of all patients”. Abbreviations: CAD-RADS, Coronary Artery Disease–Reporting and Data System; P-SIS, plaque burden based on segment involvement score.

As shown in Figure 2, higher CAD RADS categories were associated with greater plaque burden. Among patients with stenosis ≥ 50% (n = 86), 94.6% had severe (P3) or extensive (P4) plaque burden, compared with 50.7% of those without obstructive stenosis (*p* < 0.001).

### 3.3. Events According to Stenosis and Plaque Burden

Among the 41 patients who developed MACEs, 29 (70.7%) had non-obstructive CAD (CAD-RADS 1–2). Within this non-obstructive subgroup, 85.4% of events occurred in patients with severe or extensive plaque burden (P3–P4), supporting the prognostic relevance of plaque burden beyond stenosis severity alone. Overall, high-risk CAD-RADS 2.0 was more frequent among patients with MACEs than among those without events (87.8% vs. 55.4%; *p* < 0.001) (Table 3).

### 3.4. Univariable Associations with MACE

Univariable Cox analysis identified several clinical and pharmacological variables associated with MACEs, including female sex, older age, COPD, history of arrhythmias, chronic kidney disease, and treatment with beta blockers, diuretics, anticoagulants, antidiabetics, antiarrhythmics and nitrates (Table 4). Obesity was not significantly associated with MACEs. Among CCTA variables, increasing plaque burden categories showed a graded association with higher risk of MACEs. In the univariable Cox analysis, when P SIS was dichotomized (P1–P2 vs. P3–P4), severe-to-extensive plaque burden was associated with a more than 4-fold higher risk of events (HR: 4.08; 95% CI: 1.71–9.75; *p* = 0.002). In contrast, obstructive CAD defined by CAD RADS 1.0 did not show a significant independent association with MACEs.

**Figure 3:**
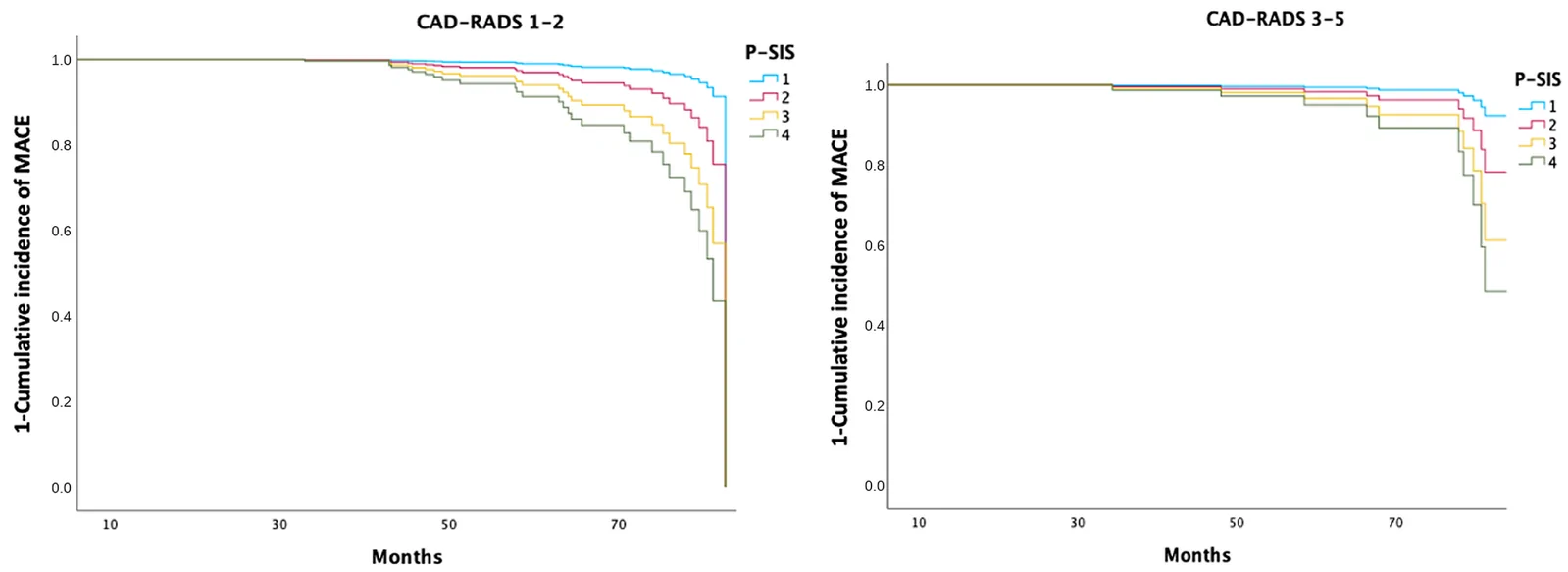
Kaplan–Meier curves of MACE-free survival according to CAD-RADS non-obstructive (1–2) versus obstructive (3–5) coronary disease. The ordinate axis indicates MACE-free survival. Abbreviations: CAD-RADS, Coronary Artery Disease–Reporting and Data System; MACE, major adverse cardiovascular event; P-SIS, plaque burden based on segment involvement score.

SIS remained significantly associated with MACEs both in the whole cohort and the cohort stratified by coronary artery stenosis degree (obstructive CAD) (Figure 3). Taking SIS = 1 as reference, in the whole cohort, more extensive coronary artery disease was associated with higher HR values (HR = 2.97, 95% CI 0.54–16.22, *p* = 0.209 for P2; HR = 5.91, 95% CI 1.28–27.38, *p* = 0.023 for P3; HR = 8.04, 95% CI 1.90–34.10, *p* = 0.005; global *p* value = 0.001). The integration of plaque burden with CAD-RADS in the high-risk CAD RADS 2.0 category was associated with a stronger increase in risk than plaque burden alone (95% CI: 1.80–11.78; *p* < 0.001).

### 3.5. Multivariable Models

In *Model 1*, age was associated with MACEs (HR per year 1.09, 95% CI 1.05–1.12; *p* < 0.001), whereas female sex (HR 1.39, 95% CI 0.67–2.86; *p* = 0.376) and significant stenosis (HR 0.79, 95% CI 0.37–1.68; *p* = 0.533) were not. In *Model 2*, age remained associated with MACEs (HR per year 1.07, 95% CI 1.03–1.10; *p* < 0.001), and elevated plaque burden was independently associated with MACEs (HR 2.60, 95% CI 1.03–6.58; *p* = 0.044), whereas female sex was not (HR 1.52, 95% CI 0.72–3.20; *p* = 0.269). In *Model 3*, age remained associated with MACEs (HR per year 1.07, 95% CI 1.03–1.10; *p* < 0.001), and the high-risk CAD-RADS 2.0 category was independently associated with MACEs (HR 2.88, 95% CI 1.07–7.75; *p* = 0.036), whereas female sex was not (HR 1.49, 95% CI 0.71–3.13; *p* = 0.295) (Table 5).

In the analysis excluding heart-failure hospitalization from the composite outcome, the high-risk CAD-RADS 2.0 category remained associated with an increased risk of events, with a similar effect size to that observed in the primary analysis, although the association did not reach conventional statistical significance, likely reflecting the lower number of events (HR 2.78, 95% CI 0.89–8.65; *p* = 0.078).

**Figure 4:**
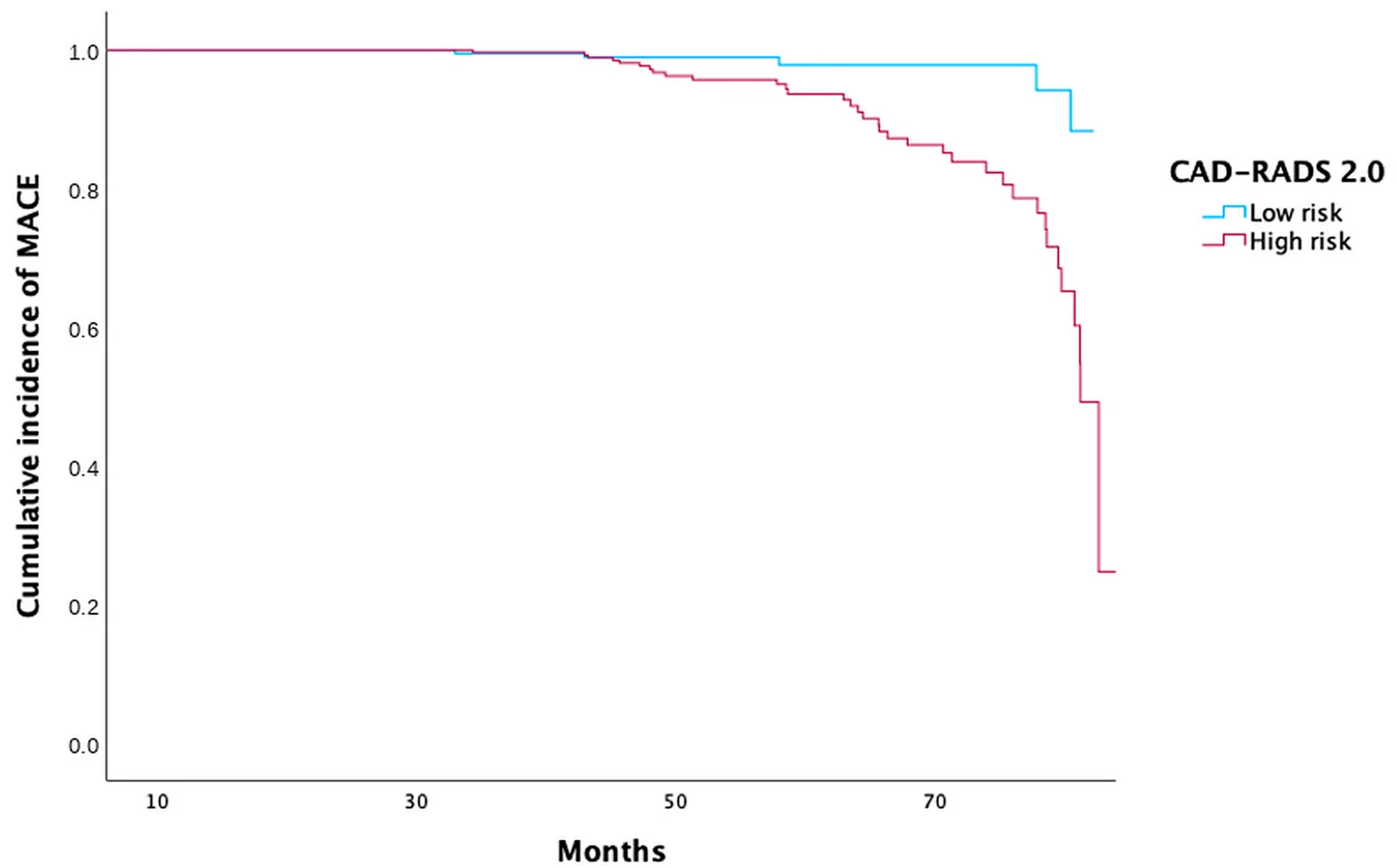
Kaplan–Meier curves of MACE-free survival in patients classified into CAD-RADS 2.0 low-risk vs. high-risk categories. Abbreviations: CAD-RADS, Coronary Artery Disease–Reporting and Data System; MACE, major adverse cardiovascular event.

Consistent with the Cox regression results, Kaplan–Meier curves showed lower MACE-free survival in patients classified as high-risk according to the CAD-RADS SIS category (Figure 4). These results provide complementary time-to-event evidence supporting the prognostic value of the high-risk classification.

**Figure 5:**
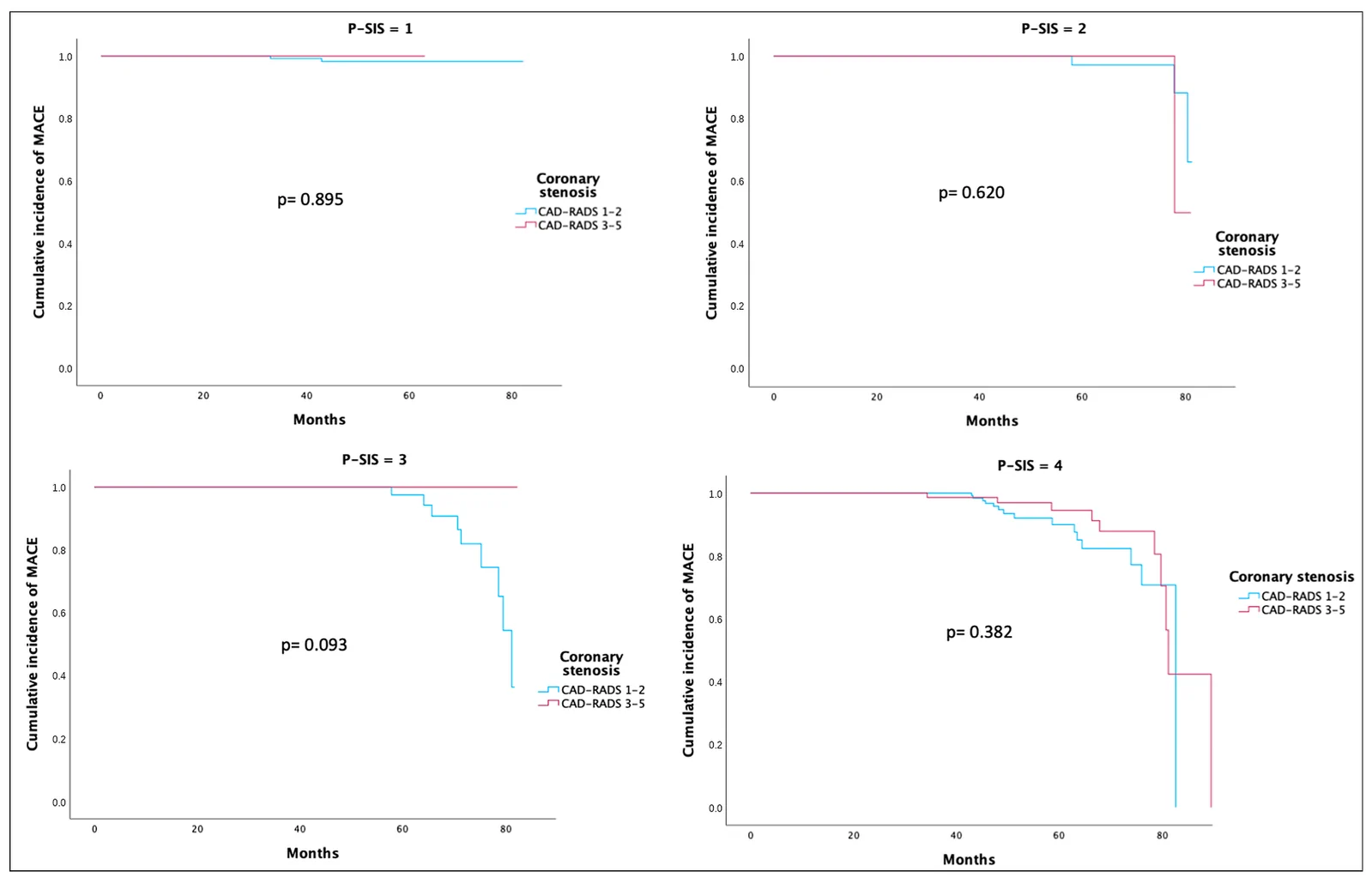
MACE-free survival according to obstructive CAD status and SIS categories. The ordinate axis indicates MACE-free survival. Abbreviations: CAD, coronary artery disease; MACE, major adverse cardiovascular event; SIS, segment involvement score.

Since plaque burden defined by SIS and prevalence of obstructive CAD were highly correlated (Figure 2), additional analyses were performed to assess their relative contribution to MACE prediction. Kaplan–Meier curves stratified by P-SIS demonstrated that patients with non-obstructive CAD but extensive plaque burden had event rates similar to those with obstructive stenosis, whereas patients with low plaque burden (P1) showed a very low incidence of MACEs throughout follow-up (Figure 5).

### 3.6. Incremental Prognostic Performance

Models 1–3 were intended to compare alternative stenosis-based, plaque burden-based, and integrated CAD-RADS 2.0 approaches, whereas the nested analysis was performed specifically to assess the incremental prognostic value of plaque burden beyond significant stenosis. Addition of plaque burden increased the Harrell C-index from 0.760 to 0.772 and significantly improved model fit (likelihood-ratio χ^2^ = 5.41; *p* = 0.020). In the extended model, plaque burden remained independently associated with MACEs (HR 2.81, 95% CI 1.10–7.19; *p* = 0.031), whereas significant stenosis was not (HR 0.72, 95% CI 0.34–1.50; *p* = 0.381).

### 3.7. Sensitivity Analysis

In the first sensitivity analysis, restricted to coronary ischemic outcomes, 16 of 563 patients (2.8%) experienced unstable angina or myocardial infarction. Events occurred in 14 of 325 patients (4.3%) in the high-risk CAD-RADS 2.0 group and in 2 of 238 patients (0.8%) in the non-high-risk group. High-risk CAD-RADS 2.0 was associated with a higher risk of coronary ischemic events in the unadjusted Cox model, although the association did not reach conventional statistical significance (HR 4.24, 95% CI 0.96–18.83; *p* = 0.057). After adjustment for age and sex, the direction of the association remained unchanged (HR 3.64, 95% CI 0.76–17.33; *p* = 0.105). In the second sensitivity analysis of MACEs defined as the composite of cardiovascular death, myocardial infarction, or unstable angina, 17 events were observed. Patients in the high-risk CAD-RADS 2.0 group had a higher risk of MACEs, although the association did not reach statistical significance after adjustment for age and sex (HR, 2.38; 95% CI, 0.63–9.04; *p* = 0.203). In the third sensitivity analysis, which included unstable angina, myocardial infarction, ischemic stroke, and cardiovascular death, 25 of 563 patients (4.4%) experienced at least one event. Events occurred in 21 of 325 patients (6.5%) in the high-risk CAD-RADS 2.0 group and in 4 of 238 patients (1.7%) in the non-high-risk group. High-risk CAD-RADS 2.0 was associated with the broader ischemic cardiovascular endpoint in the unadjusted analysis (HR 3.41, 95% CI 1.16–10.00; *p* = 0.025). After adjustment for age and sex, the association remained directionally consistent but did not reach statistical significance (HR 2.80, 95% CI 0.90–8.72; *p* = 0.076). Both sensitivity analyses should be interpreted as exploratory because of the limited number of events.

Thirty-seven patients who underwent coronary revascularization within 90 days after CCTA were reincorporated into the third sensitivity analysis, resulting in an expanded cohort of 600 patients. Three of the 37 patients (8.1%) experienced MACEs during subsequent follow-up. Thirty-six patients fulfilled the high-risk CAD-RADS 2.0 criteria, and 35 had significant stenosis. All three MACEs occurred among patients classified as high-risk CAD-RADS 2.0 and with significant stenosis. In the expanded cohort, 361 patients were classified as high-risk according to CAD-RADS 2.0. MACEs occurred in 39 of 361 high-risk patients (10.8%) and in 5 of 239 non-high-risk patients (2.1%). High-risk CAD-RADS 2.0 remained significantly associated with MACEs (HR 4.67, 95% CI 1.83–11.90; *p* = 0.001). A total of 120 patients had significant stenosis, of whom 14 (11.7%) experienced MACEs. Significant stenosis alone was not significantly associated with MACEs (HR 1.18, 95% CI 0.61–2.29; *p* = 0.619). These findings indicate that exclusion of patients undergoing early revascularization did not account for the superior prognostic association observed for the high-risk CAD-RADS 2.0 classification.

## 4. Discussion

In this cohort of patients with stable chest pain undergoing CCTA, the high-risk CAD-RADS 2.0 category was independently associated with MACEs after adjustment for age and sex, whereas significant stenosis was not. The model incorporating the high-risk classification showed slightly higher discrimination than the stenosis-based model. In the formally nested analysis, addition of the high-risk CAD-RADS SIS category to age, sex, and significant stenosis significantly improved model fit. These findings support the prognostic relevance of integrating plaque burden with stenosis severity, although the modest difference in discrimination and the limited number of events warrant cautious interpretation.

### 4.1. Prognostic Role of Plaque Burden

The first key finding of this study is that coronary plaque burden assessed by SIS provides prognostic information beyond the degree of luminal stenosis. Traditionally, CCTA interpretation has focused on identifying obstructive stenoses to guide decisions regarding revascularization. However, atherosclerosis is a diffuse disease process, and plaque burden may capture risk not fully explained by stenosis severity alone.

In our cohort, only a minority of patients (15%) presented ≥50% stenosis, whereas more than half showed severe or extensive plaque burden (P3–P4). Plaque burden remained a strong predictor of events even after adjustment for clinically relevant covariates. Moreover, patients with extensive non-obstructive disease experienced a higher event rate than those with limited plaque, supporting the concept that risk is not restricted to obstructive CAD. Conversely, as shown in the Kaplan–Meier curves, individuals with minimal plaque burden (P1) had a very low incidence of events over up to six years of follow-up, highlighting the favorable long-term prognosis associated with limited coronary atherosclerosis.

These results align with prior evidence from large registries supporting the prognostic significance of coronary plaque burden [8,13,14,15]. Large registries and multicenter cohorts have shown that extensive non-obstructive CAD carries a substantially higher risk than normal coronary arteries, with risk increasing progressively according to plaque burden and multivessel involvement [13,14,15]. Even non-obstructive CAD (1–49% stenosis) carries a worse outcome than CAD-RADS 0, with risk increasing further in the setting of three-vessel disease [13]. As a matter of fact, both semi-quantitative and fully quantitative assessments of plaque have shown strong correlations with cardiovascular events in patients with suspected or established CAD [16,17,18,19]. In addition, Shaw et al. demonstrated that non-obstructive plaque burden is independently associated with all-cause mortality, particularly among women [20].

Our findings further support the prognostic importance of overall atherosclerotic burden, showing an approximately 20% increase in event risk for each additional coronary segment containing plaque, consistent with previous reports [8]. Importantly, in our study, approximately 6% of patients with non-obstructive CAD (CAD-RADS 1–2) experienced MACEs during follow-up, underscoring that the absence of obstructive stenosis should not be equated with absence of risk.

### 4.2. Added Value of CAD-RADS 2.0

A second important finding is that CAD-RADS 2.0, through incorporation of plaque burden into the reporting framework, identified a larger proportion of patients who subsequently experienced adverse events compared with classification based on stenosis severity alone. In our study, nearly 90% of patients who experienced MACEs were classified as high-risk according to CAD-RADS 2.0, despite the relatively low prevalence of obstructive CAD in the cohort. Consequently, reliance on stenosis severity alone would have failed to identify a considerable proportion of patients who later experienced adverse outcomes, particularly among those with non-obstructive disease.

Consistent with reports from professional societies such as the Society of Cardiovascular Computed Tomography (SCCT) and the North American Society for Cardiovascular Imaging (NASCI), our findings support a paradigm shift in CCTA interpretation—from a stenosis-centered approach to one emphasizing comprehensive plaque evaluation, including burden and composition [16].

The additional prognostic contribution of plaque burden was further supported by the incremental time-to-event analyses. When severe-to-extensive plaque burden was added to a model including age, sex, and significant stenosis, overall model fit improved significantly and plaque burden remained independently associated with MACEs. Although the increase in discrimination was modest, with the Harrell C-index increasing from 0.760 to 0.772, the likelihood-ratio test demonstrated a significant improvement in model performance. These findings suggest that plaque burden provides prognostic information beyond stenosis severity alone, although its incremental contribution to individual risk discrimination after accounting for age and sex appears limited.

Importantly, the observed associations remained directionally consistent across sensitivity analyses restricted to coronary ischemic outcomes and to a broader ischemic cardiovascular endpoint comprising unstable angina, myocardial infarction, ischemic stroke, and cardiovascular death. Although statistical significance was attenuated in adjusted analyses because of the limited number of events, these findings support the robustness of the overall relationship between plaque burden and adverse cardiovascular outcomes.

Taken together, these observations support the concept that comprehensive characterization of atherosclerotic burden into CAD-RADS 2.0 may improve cardiovascular risk assessment beyond a purely stenosis-based approach.

### 4.3. Methods for the Assessment of Coronary Plaque Burden

Several approaches have been proposed to quantify coronary atherosclerotic burden, ranging from coronary artery calcification (CAC) scoring to SIS and more advanced volumetric plaque analyses. Both CAC and SIS provide prognostic information and are endorsed as options for grading plaque burden in CAD RADS 2.0, but the system does not specify which method should be preferred [9].

Prior studies have shown that CCTA-derived measures of plaque extent and composition offer incremental prognostic value beyond CAC alone in symptomatic individuals. In acute chest pain cohorts, CAC provides little additional risk stratification once CCTA findings are available [10]. A meta-analysis published in 2011 demonstrated that both the presence and extent of CAD strongly predict cardiovascular events in symptomatic patients referred for CCTA, independently of CAC and cardiovascular risk factors [15]. Similarly, three studies including more than 3000 participants demonstrated the incremental value of CCTA beyond CAC in multivariate analyses [21,22]. However, Cho et al. showed that high CAC levels, particularly scores above 300, remain powerful independent predictors of mortality in patients with known CAD, underscoring the complementary rather than interchangeable roles of CAC- and CCTA-based plaque assessment [18].

In this context, SIS offers several practical advantages. It incorporates both calcified and non-calcified plaque without additional radiation exposure or specialized software, making it readily applicable in daily practice. While it does not provide detailed information regarding plaque composition or volume, its simplicity and reproducibility make it an attractive option for standardized reporting. Emerging machine-learning-based quantitative plaque analysis may further improve risk stratification in the future, although these technologies currently remain less widely available [19].

### 4.4. Limitations

Several limitations should be considered when interpreting these findings. First, it is a retrospective, single-center analysis, which may introduce selection bias and limit generalizability. Second, management decisions, pharmacological treatments and lifestyle interventions were not protocolized and may have influenced outcomes. Third, some patients were excluded because of missing data or early revascularization, which could have attenuated event rates in higher stenosis categories. Fourth, the composite endpoint was relatively broad and included heart-failure hospitalization, cerebrovascular events, and mortality in addition to coronary ischemic outcomes. Although sensitivity analyses restricted to ischemic cardiovascular events yielded qualitatively similar findings, the limited number of events reduced statistical precision. Fifth, qualitative high-risk plaque characteristics such as low-attenuation plaque, positive remodeling, and the napkin-ring sign were not systematically evaluated and may provide complementary prognostic information beyond plaque burden alone. Finally, only 41 MACEs occurred during follow-up. Accordingly, the multivariable strategy was revised to use clinically justified parsimonious models, and no automated or stepwise variable selection procedure was applied. This revised modelling strategy was introduced during manuscript revision and was not prespecified in the original study protocol. Furthermore, the prognostic models were not externally validated, and the incremental improvement in discrimination beyond age, sex, and stenosis severity was modest. Consequently, the findings should be interpreted cautiously and considered hypothesis-generating until confirmed in larger prospective multicenter cohorts.

Patients undergoing coronary revascularization within 90 days after CCTA were excluded from the primary analysis to minimize treatment-related modification of subsequent risk. Although this approach may reduce the representation of patients with severe obstructive CAD, a sensitivity analysis including these patients yielded results that were consistent with the primary findings.

## 5. Conclusions

In patients with stable chest pain undergoing CCTA, severe-to-extensive plaque burden was associated with adverse cardiovascular outcomes beyond stenosis severity alone. The high-risk CAD-RADS 2.0 category identified patients at increased risk of MACEs, whereas formal incremental analyses demonstrated that plaque burden provided prognostic information beyond age, sex, and significant stenosis. Larger prospective studies with external validation are needed to determine whether systematic incorporation of plaque burden assessment into routine CCTA reporting improves risk stratification, clinical decision-making, and patient outcomes.

## Figures and Tables

**Table 1:** Baseline characteristics of the cohort according to MACE occurrence. Abbreviations: CAD, coronary artery disease; COPD, chronic obstructive pulmonary disease; MACE, major adverse cardiovascular event.

Variable	MACE N = 41	No MACE N = 522	*p*-Value
Sex (male/female)	17 (41.5)/24 (58.5)	347 (66.5)/175 (33.5)	<0.001
Age ^a^	74 (68.5–81.5)	62 (53–69)	<0.001
Arterial hypertension	26 (63.4)	246 (47.1)	0.029
Dyslipidemia	29 (70.7)	310 (59.4)	0.153
Obesity ^b^	7 (17.1)	97 (18.6)	0.833
Smoking habit *	22 (53.7)	289 (55.4)	0.696
Hyperuricemia	7 (17.1)	54 (10.4)	0.184
Family history of CAD	11 (26.8)	222 (42.5)	0.047
Diabetes	11 (26.8)	58 (11.1)	0.003
History of arrhythmias	16 (39)	84 (16.1)	<0.001
History of stroke	4 (12.8)	17 (3.3)	0.035
Peripheral artery disease	2 (4.9)	16 (3.1)	0.525
Chronic kidney disease	4 (9.8)	15 (2.9)	0.019
COPD	5 (14.2)	17 (3.3)	0.008

Unless otherwise specified, data expressed are as number of patients with percentage in parentheses. ^a^ Data are expressed as median and interquartile range. ^b^ Obesity was defined as a body mass index ≥ 30 kg/m^2^ and was analyzed as a categorical variable. * Former smoker or current smoker.

**Table 2:** Pharmacological treatment of the cohort, stratified by the presence of MACEs. Abbreviations: BPH, benign prostatic hyperplasia; MACE, major adverse cardiovascular event.

Variable	MACE N = 41	No MACE N = 522	*p*-Value
Antihypertensives	25 (61)	224 (42.9)	0.023
Beta-blockers	18 (43.9)	115 (22)	0.001
Diuretics	28 (68.3)	92 (17.6)	<0.001
Lipid-lowering agents	23 (56.1)	244 (46.7)	0.248
Antidiabetics	9 (22)	62 (11.9)	0.061
Antiplatelet agents	13 (31.7)	94 (18)	0.032
Anticoagulants	15 (36.6)	61 (11.7)	<0.001
Antiarrhythmics	6 (14.6)	20 (3.4)	0.002
Nitrates	6 (14.6)	17 (3.3)	<0.001
Allopurinol	4 (9.8)	24 (4.6)	0.143
Bronchodilators	5 (12.2)	32 (6.1)	0.132
BPH drugs	1 (2.4)	38 (7.3)	0.240

BPH: benign prostatic hyperplasia.

**Table 3:** CCTA findings of the cohort, stratified by the presence of MACEs. Abbreviations: CAD, coronary artery disease; CAD-RADS, Coronary Artery Disease–Reporting and Data System; CCTA, coronary computed tomography angiography; MACE, major adverse cardiovascular event; SIS, segment involvement score.

Variable	MACE N = 41 n (%)	No MACE N = 522 n (%)	*p*-Value
CAD-RADS category			
1	12 (29.3)	339 (64.9)	<0.001
2	17 (41.5)	109 (20.9)	
3	7 (17.1)	37 (7.1)	
4	3 (7.3)	29 (5.6)	
5	2 (4.9)	8 (1.5)	
Plaque burden grade (SIS)			
P1	2 (4.9)	133 (25.5)	0.002
P2	4 (9.8)	101 (19.3)	
P3	9 (22)	75 (14.4)	
P4	26 (63.4)	213 (40.8)	
Obstructive CAD according to CAD-RADS 1	12 (29.3)	74 (14.1)	0.023
High-risk patients according to CAD-RADS 2.0	36 (87.8)	289 (55.4)	<0.001

**Table 4:** Univariable Cox regression analyses for predicting MACEs. Abbreviations: CAD, coronary artery disease; CAD-RADS, Coronary Artery Disease–Reporting and Data System; CCTA, coronary computed tomography angiography; CI, confidence interval; COPD, chronic obstructive pulmonary disease; HR, hazard ratio; MACE, major adverse cardiovascular event.

Variable	Hazard Ratio (HR)	Confidence Interval (CI)	*p*-Value
**Clinical variables**			
Sex (male as reference)	2.37	1.24–4.54	0.009
Age ^a^	1.09	1.06–1.12	<0.001
Arterial hypertension	1.83	0.94–3.58	0.076
Dyslipidemia	1.49	0.75–2.95	0.251
Obesity	1.13	0.47–2.73	0.785
Smoking habit *	1.08	0.58–2.03	0.811
Hyperuricemia	2.02	0.89–4.60	0.093
Family history of CAD	0.82	0.41–1.65	0.821
Diabetes	1.99	0.99–4.00	0.054
History of arrhythmias	3.71	1.93–7.15	<0.001
History of stroke	2.65	0.94–7.47	0.066
Peripheral artery disease	1.17	0.28–4.89	0.832
Chronic kidney disease	3.20	1.13–9.08	0.029
COPD	3.87	1.61–9.29	0.003
**Pharmacological variables**			
Antihypertensives	1.73	0.97–3.43	0.083
Beta-blockers	2.60	1.39–4.86	0.003
Diuretics	6.90	3.50–13.62	<0.001
Lipid-lowering agents	1.39	0.74–2.62	0.305
Antidiabetics	3.82	1.79–8.16	<0.001
Antiplatelet agents	1.57	0.81–3.07	0.184
Anticoagulants	5.12	2.68–9.79	<0.001
Antiarrhythmics	2.82	1.18–6.76	0.020
Nitrates	3.27	1.36–7.86	0.008
Allopurinol	2.25	0.80–6.33	0.125
Bronchodilators	2.03	0.79–5.19	0.149
BPH drugs	0.50	0.07–3.62	0.488
**CCTA variables**			
CAD-RADS category			
1	reference	reference	
2	0.58	0.30–1.14	0.116
3	1.92	1.03–3.58	0.040
4	1.36	0.62–2.95	0.441
5	0.96	0.35–2.67	0.943
Plaque burden grade			
P1	reference	reference	
P2	2.97	0.54–16.23	0.209
P3	5.91	1.28–27.38	0.023
P4	8.04	1.90–34.1	0.005
Obstructive CAD according to CAD-RADS 1	1.05	0.51–2.16	0.906
Plaque burden (P1-P2 vs. P3-P4)	4.08	1.71–9.75	0.002
High-risk patients according to CAD-RADS 2.0	4.60	1.80–11.78	0.001

^a^ Male as reference. * Non-smoker as reference.

**Table 5:** Parsimonious Cox regression analyses for predicting MACEs. Abbreviations: CAD-RADS, Coronary Artery Disease–Reporting and Data System; CI, confidence interval; HR, hazard ratio.

Variable	HR	95% CI	*p* Value
**Model 1: age, sex, and significant stenosis**			
Age, per year	1.09	1.05–1.12	<0.001
Female sex	1.39	0.67–2.86	0.376
Significant stenosis	0.79	0.37–1.68	0.533
**Model 2: age, sex, and elevated plaque burden**			
Age, per year	1.07	1.03–1.10	<0.001
Female sex	1.52	0.72–3.20	0.269
Elevated plaque burden	2.60	1.03–6.58	0.044
**Model 3: age, sex, and high-risk CAD-RADS 2.0 category**			
Age, per year	1.07	1.03–1.10	<0.001
Female sex	1.49	0.71–3.13	0.295
High-risk CAD-RADS 2.0 category	2.88	1.07–7.75	0.036

## Data Availability

The data presented in this study are available on request from the corresponding author, because the data are not publicly available due to due to privacy or ethical restrictions.

## Abbreviations

The following abbreviations are used in this manuscript:

CCTA	coronary computed tomography angiography
CAD-RADS	coronary artery disease–reporting and data system
CAD	coronary artery disease
MACE	major adverse cardiovascular events
SIS	segment involvement score
ADMIRE	advanced modeled iterative reconstruction
CI	confidence interval
HR	hazard ratio
IQR	interquartile range
COPD	chronic obstructive pulmonary disease
BPH	benign prostatic hyperplasia
CKD	chronic kidney disease
CAC	coronary artery calcification
BMI	body mass index
